# Tracheal Ring Formation

**DOI:** 10.3389/fcell.2022.900447

**Published:** 2022-04-25

**Authors:** Dagmar Iber, Malte Mederacke

**Affiliations:** ^1^ Department of Biosystems Science and Engineering, ETH Zurich, Basel, Switzerland; ^2^ Swiss Institute of Bioinformatics, Basel, Switzerland

**Keywords:** trachea, cartilage rings, symmetry break, SOX9, Turing pattern, chemotaxis, differential adhesion, differential growth

## Abstract

The trachea is a long tube that enables air passage between the larynx and the bronchi. C-shaped cartilage rings on the ventral side stabilise the structure. On its esophagus-facing dorsal side, deformable smooth muscle facilitates the passage of food in the esophagus. While the symmetry break along the dorsal-ventral axis is well understood, the molecular mechanism that results in the periodic *Sox9* expression pattern that translates into the cartilage rings has remained elusive. Here, we review the molecular regulatory interactions that have been elucidated, and discuss possible patterning mechanisms. Understanding the principles of self-organisation is important, both to define biomedical interventions and to enable tissue engineering.

## 1 Introduction

**FIGURE 1:**
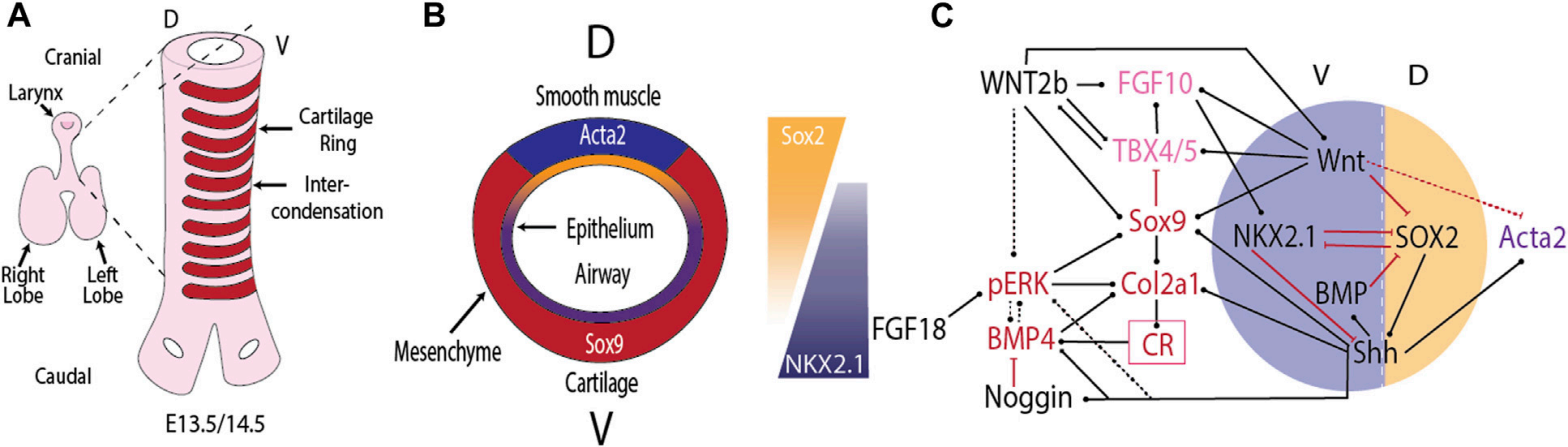
Tracheal cartilage ring formation. **(A)** Cartilage rings (red) emerge in the mesenchyme on the ventral (V) side of the trachea. **(B)** Cross-section of the developing trachea. **(C)** Regulatory interactions that control the emergence of cartilage rings (CR) in the ventral and airway smooth muscle in the dorsal (D) tracheal mesenchyme. Black arrows indicate positive regulation, red arrows negative regulation. For details see text.

The trachea is a long (6 mm in mice, 10–15 cm in human), almost cylindrical tube that serves as a passage of air to the bronchial system Kishimoto and Morimoto (2021). Its wide diameter (1.5 mm in mice, 2–3 cm in human) poses little resistance to air flow. C-shaped cartilage rings on its ventral side prevent the collapse or obstruction of the tube (Figure 1A). Smooth muscle on the dorsal side allows for the expansion of the adjacent esophagus during the consumption of food or liquid. The separation into distinct domains that form cartilage and smooth muscles, and the subsequent emergence of cartilage rings reflects two separate symmetry breaks. While the first one is well understood, the molecular mechanism behind the second has remained elusive. In the following, we will discuss the regulatory interactions that are involved in these symmetry breaks.

## 2 Dorsal-Ventral Polarity

The separation of cartilage and smooth muscles domains follows the already established dorsal-ventral polarity. Fibroblastic growth factor (FGF) from the cardiac mesoderm induces the *Nkx2.1*-expressing lung field on the ventral side of the mouse foregut Serls et al. (2005). Bone morphogenetic protein 4 (*Bmp4*) expression is restricted to the ventral foregut from early stages (E8.5) Li et al. (2008), and the BMP antagonist NOGGIN is secreted from the dorsally located notochord Fausett et al. (2014). BMP4 signalling surpresses SRY (sex determining region Y)-box transcription factor (*Sox*)2 expression in the ventral foregut Domyan et al. (2011). Mutual repression between NKX2.1, which is restricted to the ventral foregut endoderm, and SOX2, which is expressed in the dorsal foregut endoderm, defines the border between the trachea, and the future esophagus Que et al. (2007). NKX2.1 directly represses *Efnb2*, which establishes an EPH/EPHRIN boundary that results in the physical separation of tracheal and esophageal cells Lewis et al. (2022). *Nkx2.1* null mice, and endodermal mutants for the BMP type I receptor genes *Bmpr1a* and *Bmpr1b* upregulate *Sox2* and form a continuous ring of smooth muscle and no cartilage rings Que et al. (2007); Minoo et al. (1999); Yuan et al. (2000); Li et al. (2008); Domyan et al. (2011). Conditional ablation of *Bmp4* from the foregut endoderm from E8.5 and from the mesenchyme by E9.5 does not prevent the ventral expression of *Nkx2.1* at E9.5, but by E12.5 *Nkx2.1* is absent, and expression of the cartilage marker Collagen Type II Alpha 1 Chain (*Col2a1*) is not observed Li et al. (2008).

Once the trachea has split from the future esophagus, it maintains the dorsal-ventral polarity, with *Nkx2.1* expression restricted to the ventral side, and *Sox2* and Sonic Hedgehog (*Shh*) expression higher on the dorsal side (Figure 1B). This polarity is observed also in mutants (*Noggin* null) that fail to split the tubes Que et al. (2006). The epithelial dorsal–ventral polarity translates into a mesenchymal polarity. Mesenchymal cells derived from the splanchnic mesoderm, positioned ventral to the developing tracheal tube express the transcription factor *Sox9* as early as E10.5 Hines et al. (2013). From E11.5, dorsal mesenchymal cells express *Acta2*, a smooth muscle marker Hines et al. (2013). Removal of *Sox9* or a key smooth muscle gene does not alter the expression domain of the other in the trachea Hines et al. (2013). The spatial restriction is thus not maintained by mutual repression between SOX9 and smooth muscle genes. Rather, signals from the tracheal epithelium appear important for the spatial restriction in the mesenchyme. Blockage of WNT secretion from the tracheal epithelium in *Wls* conditional mutants blocks *Sox9* expression and results in smooth muscle formation also on the ventral side Snowball et al. (2015); Kishimoto et al. (2020). Epithelial WNT secretion thus seems to be required in translating the epithelial polarity to the mesenchyme. Canonical WNT signalling appears to be important in both layers as conditional removal of *β*-catenin in either the epithelium (*Shh-Cre* driven) or mesenchyme (*Dermo1-Cre* driven) results in loss of mesenchymal expression of the chondrogenic factor *Tbx4* Kishimoto et al. (2020). In *Shh* null mice, the ventral restriction of *Sox9* expression is lost, and until E13.5, *Sox9* is transiently weakly expressed in a circumferential expression pattern on both the dorsal and ventral sides Park et al. (2010). Even though *Shh* is expressed more strongly dorsally, overexpression of *Shh* does not affect the relative cartilage and smooth muscle domains Sala et al. (2011). Addition of BMP4 or Noggin to lung explant cultures induces patches of cartilage formation and *Sox9* and *Bmp4* expression around the entire tracheal epithelium Park et al. (2010).

In summary, the separation of the smooth muscle and cartilage domains along the dorsal-ventral axis is controlled by the already existing embryonic dorsal-ventral polarity. The dorsal-ventral polarity is first induced along the epithelial tube, and later translated to the mesenchyme via diffusible morphogens.

## 3 Emergence of Periodic Patterns Along the Trachea

The positions of the future cartilage rings in the ventral tracheal mesenchyme first become apparent between embryonic day (E)12.75 and E13 as periodic patterns in *Sox9* and type II collagen (*Col2a1*) expression Miller et al. (2004); Elluru et al. (2009); Park et al. (2010); Sala et al. (2011); Hines et al. (2013); Turcatel et al. (2013); Boucherat et al. (2014); Young et al. (2020). Lineage tracing experiments with Col2a1-mTmG mice show that *Col2a1*-expressing cells do not transdifferentiate into non-cartilage cells Young et al. (2020). Rather, the *Col2a1*-expressing cells condense in the cartilage rings, and the intervening space becomes filled by other mesenchymal cells. The *Col2a1* gene encodes the pro-alpha1 (II) chain component of type II collagen, which is primarily found in cartilage. At E11.5, collagen type II is restricted to the lamina propria on the ventral side of the trachea Sala et al. (2011). By E12.5, collagen type II has spread into the ventral mesenchyme, but no staining is observed in the ventral half of the ventral mesenchyme. By E13.5, collagen type II-positive condensations are observed. At the same time, phosphorylated extracellular signal-regulated kinase (ERK) is found mainly on the boundary of the cartilage condensations and at lower levels between the condensations, and is largely absent from the condensations Yoshida et al. (2020). Expression of the SHH receptor *Ptch1* appears to be restricted to the nascent cartilage condensations from E13.5 Miller et al. (2004). In parallel, the expression of *Shh* assumes a periodic pattern on the ventral, but not on the dorsal side of the tracheal epithelial tube Sala et al. (2011). Around E13.5 or slightly after, *Tbx5* disappears from the cartilage condensations Tiozzo et al. (2009); Arora et al. (2012). From E14.5, *Fgf10* expression becomes restricted in between the nascent cartilage condensations, but its receptor *Fgfr2b* remains uniformly expressed in the epithelial tube Sala et al. (2011). The cartilage condensations secrete BMP4 by E17.5 Park et al. (2010); it is not known at what time the spatial restriction of *Bmp4* emerges.

## 4 Mutants Without Cartilage Rings

Functional genetics can help to identify the components of the core mechanism as their null mutations should result in the loss of cartilage rings. In the following, we will focus on mutants that do not show any periodic *Sox9*/*Col2a1* expression patterns or tracheal cartilage ring formation, even though the trachea forms with correct dorsal-ventral polarity. This analysis thus necessarily excludes potential core components that are involved also in processes upstream of periodic pattern formation as their contribution to periodic patterning cannot be evaluated by this approach. The following mouse mutants have so far been reported that lack tracheal cartilage rings, even though the trachea forms with correct dorsal-ventral polarity: *Shh* null Miller et al. (2004); Park et al. (2010), *Sox9* null Hines et al. (2013); Turcatel et al. (2013), mesenchymal *Mek1*/*Mek2* removal Boucherat et al. (2014), and endodermal *Wls* removal Snowball et al. (2015). Finally, in mouse double mutants of R-spondin2 and lipoprotein receptor related protein 6 (*Rspo2Tg/Tg*;*Lrp6* ^−/−^) tracheal rings were absent on the shortened tracheal structure Bell et al. (2008), and *Dermo1-Cre*-driven conditional removal of *β*-catenin, a core component of canonical WNT signalling, result in loss of mesodermal *Tbx4*, impaired mesenchymal growth, and lack of cartilage rings at E16.5 Kishimoto et al. (2020). A mutation in human FGFR2 (S351C) prevents visible tracheal ring formation, but the cartilaginous tracheal sleeve still forms Gonzales et al. (2005). Alternative mRNA splicing in one of the extracellular immunoglobulin (Ig)-like domains results in different FGF receptor isoforms, known as FGFR (IIIb), and FGFR (IIIc) Johnson and Williams (1993). The isoforms differ in their ligand specificity and expression pattern. FGFR2(IIIb) is produced predominantly in epithelial cells and binds to FGF7 and FGF10, while FGFR2(IIIc) is found in the mesenchyme. *Fgfr2b* and *Fgf10* null mice have a different phenotype from that reported for human FGFR2 (S351C) in that they develop shorter tracheas with 6–8 distorted cartilage rings Min et al. (1998); Sekine et al. (1999); Sala et al. (2011), suggesting that the phenotype of human FGFR2 (S351C) results from defects in the mesenchymal isoform. Ectopic mesenchymal expression of *FgfR2b* in *FgfR2c* heterozygous mouse mutants results in overgrowth of the tracheal rings and absence of noncartilaginous mesenchyme Tiozzo et al. (2009).

BMP4 and its antagonist NOGGIN can both rescue cartilage formation as well as *Sox9* and *Bmp4* expression in *Shh* null lung explants, but cartilage formation is then no longer restricted to the ventral side; it has not been reported whether periodic patterns are obtained Park et al. (2010). BMP4 and Noggin induce additional cartilage formation also in wildtype lungs, and cartilage then forms also on the dorsal side. In *Bmp4* conditional mutants, *Nkx2.1* is restricted to the ventral side at E9.5, but is lost by E12.5, and no *Col2a1* expression and cartilage ring formation is observed Li et al. (2008). Mice with inactivated *Bmpr1b* and *Sox2*, and a SHH-driven endodermal conditional knockout of *Bmpr1a* develop a ventral NKX2.1 domain that forms disorganized isolated cartilage pieces/nodules, but not rings at later stages Domyan et al. (2011). Epithelial BMP signalling thus appears not to be necessary for the emergence of the periodic cartilage pattern. It is unclear whether mesenchymal BMP signalling is required for periodic cartilage formation as a combined mesenchymal knockout of *Bmpr1a* and *Bmpr1b* has so far not been reported.

While perturbations in many other pathways affect tracheal ring formation or tracheal growth, no other pathway has been described that is necessary for cartilage ring formation once the tracheal mesenchyme has emerged Iber (2021).

## 5 Control of Cartilage Ring Formation

SOX9 controls all steps of the cartilage differentiation process, and is a necessary factor for cartilage ring formation such that cartilage rings are absent in mesenchymal *Sox9* knockout mice Hines et al. (2013); Turcatel et al. (2013). If doxycycline-driven *Sox9* removal is stopped at E13.5, then some cartilage nodules are observed by E18.5 in the most proximal part Turcatel et al. (2013). Progressively more distal nodules are observed if doxycycline induction is stopped already at E12.5 or E11.5 Turcatel et al. (2013). Secretion of endodermal WNT via WLS is required for mesenchymal *Sox9* expression Snowball et al. (2015), and *Sox9* expression appears strongly reduced or absent in *Rspo2Tg/Tg* mutant tracheal mesenchyme Bell et al. (2008). More generally, epithelial WNT ligands including WNT7b and WNT5a activate WNT/*β*-catenin in the mesenchyme of the developing trachea to influence expression of chondrogenic factors including *Tbx4*, *Tbx5*, *Msx1*, *Msx2*, *Sox9*, and *Col2a1* Snowball et al. (2015); Kishimoto et al. (2020). SHH signalling induces the expression of *Wnt5a* and its receptor *Ror2*, and ablation of *Wnt5a* or its receptor *Ror2* results in shorter trachea with fewer cartilage rings Li et al. (2002); Oishi et al. (2003). Deletion of *Wnt7b*, expressed by the respiratory epithelium and known to mediate Wnt/*β*-catenin signaling, does not affect trachea length or width, but results in incomplete cartilaginous rings Rajagopal et al. (2008). Deletion of *Wnt4* does not affect tracheal length, but results in 12 distorted rather than 14 tracheal rings, and results in reduced *Sox9* and increased *Fgf10* expression at 13.5 Caprioli et al. (2015).

SOX9 is a direct regulator of *Col2a1* expression (Figure 1C), a necessary factor for cartilage formation Rockich et al. (2013); Boucherat et al. (2014). Despite the direct regulation, the expression of *Sox9* and *Col2a1* is largely independently regulated. Thus, *Col2a1* rings emerge in *Tbx4*/*Tbx5* conditional mutants even though *Sox9* expression is rather weak, and *Sox9* rings are barely visible Arora et al. (2012). Vice versa, *Col2a1* is absent in *Shh* null mice, even though *Sox9* is expressed until E13.5 Park et al. (2010). While *Sox9* is weakly expressed in *Shh* null mice until E13.5, the ventral restriction of *Sox9* expression is lost, and *Sox9* expression is completely lost by E15 Pepicelli et al. (1998); Park et al. (2010). One group reported disorganised cartilage ring formation in *Shh* null mice Pepicelli et al. (1998), but other groups failed to observe cartilage rings Park et al. (2010); Miller et al. (2004).

A study in chondrocytes showed that SOX9-GLI directly and cooperatively regulate many genes such as *Sox9*, *Col2a1*, *Ptch1*, *Gli1*, *Gli2*, *Fgfr3*, *Igf1r,* and *Bmp6* Tan et al. (2018). SHH signalling may thus engage in a positive feedback with SOX9. SHH signalling represses *Fgf10* expression, and *Fgf10* disappears from the mesenchymal condensations by E14.5 Bellusci et al. (1997); Park et al. (1998); Abler et al. (2009). In the absence of *Sox9*, the expression of *Fgf10*, *Tbx4*, and *Tbx5* remains uniform Turcatel et al. (2013). Conditional removal of *Tbx4*, and *Tbx5* has similar effects on trachea development as removal of *Fgf10*, but, even though TBX4/5 promote *Fgf10* expression Cebra-Thomas et al. (2003); Sakiyama et al. (2003), they appear to act also independently of FGF10 during trachea development Arora et al. (2012). *Bmp4*, *Wnt2/2b*, and *Sox9* are strongly reduced in *Tbx4/5* conditional mutants, but *Col2a1* levels appear normal.

Mesenchymal removal of *Mek1*/*Mek2* results in a thinner trachea with continuous, but lower *Sox9* expression at E14.5 and without cartilage rings by E18.5 Boucherat et al. (2014). Epithelial removal of *Mek1*/*Mek2* results in a shorter trachea with fewer cartilage rings. Culturing lung explants with PD0325901, an inhibitor for MEK, results in increased *Col2a1* expression and a widening of the cartilage condensations, but has no impact on *Sox9* expression Yoshida et al. (2020). This is consistent with reports in other systems that show that mesenchymal phosphorylated ERK (a kinase downstream of MEK) opposes cartilage formation Oh et al. (2000); Ibarra et al. (2021). The differences between the culture experiments and the mesenchymal knockouts likely reflect differences in dosage and spatial restriction.

Despite its importance for cartilage ring formation, the upstream regulators of the MEK/ERK cascasde have remained elusive. FGFs signal via ERK, and overexpression of *Fgf18* results in abnormal tracheal cartilage formation Elluru et al. (2009), but the knockout of *Fgf18* does not result in a tracheal phenotype Usui et al. (2004). The FGF10 receptor, FGFR2b, is restricted to the tracheal epithelium Sala et al. (2011), and can therefore not trigger mesenchymal ERK activation. BMP4 appears to be the main inducer of ERK1/2 activation in the E9.25 ventral endoderm and mesoderm Li et al. (2008), but it is not known whether it remains so also at later stages when mesenchymal condensations form (E12.5-E13.5). A cell culture study concluded that BMP2 induces *Sox9* transcription mainly via p38 MAP Kinase (MAPK), while regulating SOX9 transcription factor activity via pSMAD1/5/8 and p38 Pan et al. (2008). A number of other mechanisms has been found to activate ERK in other contexts. For one, mesenchymal WNT signalling has recently been shown to activate pERK in the cranial mesenchyme, which then blocks *Sox9* and *Col2a* expression and cartilage formation Ibarra et al. (2021). Non-canonical SHH signaling has been suggested to trigger calcium-induced extracellular signal-regulated kinases (ERK) activation Robbins et al. (2012); Carballo et al. (2018). ERK may also respond to pressure and/or curvature, as reported for the lung epithelium Hirashima and Matsuda (2021). In epithelial cells from the mammary gland, ERK activity has been found sensitive to the stiffness of the surrounding matrix Farahani et al. (2021). Whether any of this plays a role in the tracheal mesenchyme is not known.

Interestingly, upon conditional removal of Myorcardin, the cartilage rings fail to expand towards the dorsal side, and the trachael lumen is reduced Young et al. (2020). Considering that smooth muscles and peristalsis are undetectable, and the expression of two BMP inhibitors is decreased and pSMAD signalling is increased in the mutants, this could be the consequence of either mechanical and/or signalling defects.

In summary, WNT signalling (WLS, R-spondin2/LRP6) is essential for mesenchymal *Sox9* expression, and SOX9 is essential for cartilage formation. *Sox9* is still expressed weakly in *Shh* and mesenchymal *Mek1*/*Mek2* mutants, but fails to organise into rings. As such, SHH and MEK1/2 are either part of the core mechanism that results in periodic *Sox9* patterning, or periodic patterning fails because *Sox9* expression is too weak in those mutants. Myocardin, a master regulatory of smooth muscle differentiation, is necessary for the dorsal expansion of the nascent cartilage rings to their characteristic C-shape. But what leads to the periodic *Sox9* pattern?

## 6 Candidate Mechanisms for Periodic Pattern Formation

**FIGURE 2:**
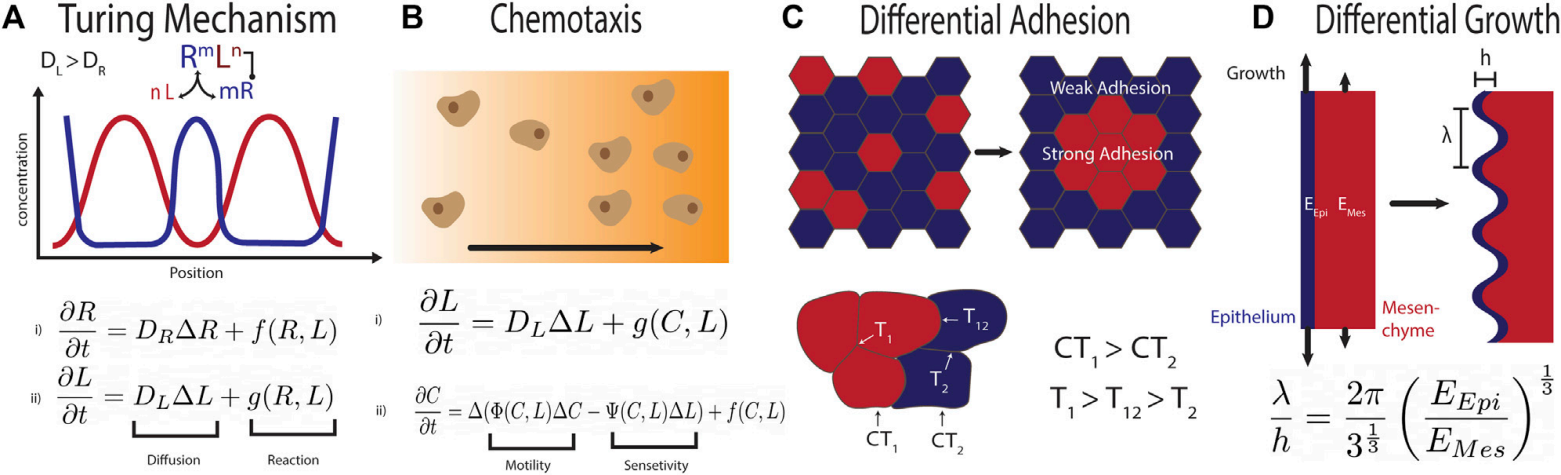
**(A)** Repetitive patterns can emerge via a Turing mechanism when two regulatory factors that diffuse at different speeds engage in a negative feedback Turing (1952). In case of the ligand-receptor based Turing mechanism, the binding of the rapidly diffusing ligand (L) to the slowly diffusing receptor (R) results in up-regulation of the receptor and removal of the ligand. This system can be modelled with two coupled partial differential reaction diffusion equations. This system can yield a large variety of patterns, dependent on the reaction parameters and the tissue geometry. **(B)** Chemotaxis can result in periodic patterning when motile cells (C) produce and consume the diffusible chemoattractant or chemorepellent (*L*), as modelled for instance by the Keller-Segel model Keller and Segel (1971). **(C)** Differential adhesion between the blue and the red cells can result in periodic pattern formation. The mixture of cells is dependent on their relative surface tension (CT_1_ for the red, CT_2_ for the blue population). This results in three different relative surface tensions (T_1_ between red cells, T_2_ between blue and T_12_ between the two populations) Canty et al. (2017). To achieve separation in the displayed form CT_1_ must be larger than CT_2_ and T_1_ larger than T_12_ which is larger than T_2_. **(D)** Differential growth of two adjacent tissue layers can result in buckling. The wavelength *λ* of the periodic pattern depends on the thickness, *h*, of the expanding epithelial layer (blue layer), and the relative Young modulus *E*_*Epi*_/*E*_*Mes*_ of epithelium (blue) and mesenchyme (red) Sultan and Boudaoud (2008).

A wide range of chemical and/or mechanical instabilities can result in biological pattern formation. The Swift-Hohenberg equation has been shown to recapitulate the complex tracheal cartilage pattern also at the tracheobranchial juncture, if coupled with a gradient to achieve the correct stripe orientation Kingsley et al. (2018). While the Swift-Hohenberg equation can be derived from fundamental equations for the Rayleigh-Benard convection in an heated fluid Swift and Hohenberg (1977), it has remained difficult to find a mechanistic explanation for the forth-order spatial derivative in biology Oza et al. (2016). Given its patterning versatility, Turing mechanisms Turing (1952) (Figure 2A) have been proposed for a large number of biological patterning processes, including tracheal cartilage ring formation Sala et al. (2011); Kingsley et al. (2018). While the mathematical properties of Turing mechanisms are well understood Murray (2003), and Turing patterns have been confirmed in chemical reaction systems Horvath et al. (2009), the molecular mechanism behind biological Turing mechanisms remains unknown. The experimental validation of proposed molecular implementations of Turing mechanisms remains impossible as kinetic parameters cannot be measured reliably in biological tissues and pattern likeness is insufficient proof. As such, only the experimental rejection of Turing mechanisms is possible to date. A well-known example are the stripes in the *Drosophila* blastoderm, which were initially accounted to a Turing mechanism, but have since been shown to result from cross-repressive transcription factor cascades downstream of opposing morphogen gradients Meinhardt (1986); Lacalli et al. (1988); Akam (1989); Jaeger (2011). In many other complex, stereotypic patterning systems, Turing models have remained the only candidate mechanism that is consistent with the experimental data. In its simplest form, Turing patterns require a negative feedback between at least two components that diffuse at different speed. Turing patterns can be obtained also with a single morphogen or growth factor if its binding to the cell-bound receptor upregulates the receptor concentration (Figure 2A), as is the case for SHH, FGF10, and BMP Menshykau et al. (2012); Badugu et al. (2012); Celliere et al. (2012); Kurics et al. (2014); Menshykau et al. (2014). Candidate networks for Turing models that yield periodic cartilage patterns have been studied extensively in limb development, where the cartilage condensations mark future digits and phalanges Iber and Germann (2014). The patterns in wildtype and perturbed conditions could be explained with a variety of biological mechanisms, including a 3-node network composed of BMP, SOX9, and WNT Raspopovic et al. (2014), a negative feedback between TGF-*β* and either the extracellular matrix (ECM) or TGF-*β* antagonists Zhu et al. (2010), and the interaction between BMPs and its receptor Badugu et al. (2012). These mechanisms have so far not been explored in the trachea. Mesodermal *β*-catenin appears necessary also for tracheal *Sox9* expression Kishimoto et al. (2020), but this makes it difficult to assess the role of WNT signalling in the subsequent periodic patterning of SOX9. In case of a ligand-receptor-based Turing mechanism, the receptor would have to be upregulated in the tracheal cartilage condensations. This has indeed been reported for the SHH receptor PTCH1 Miller et al. (2004). The expression patterns of *Bmpr1a* and *Bmpr1b* are not known. Unlike in lung branching morphogenesis Menshykau et al. (2014); Kurics et al. (2014), FGF10 is unlikely to be part of the core Turing mechanism as its receptor remains uniformly expressed in the tracheal epithelium, and periodic collagen type II patterns are still observed in *Fgf10* mice, if delayed and less uniformly shaped compared to the wildtype Sala et al. (2011). In case of a SHH-based ligand-receptor-based Turing mechanism, uniform SHH signalling on the dorsal side of the trachea could be explained with the higher *Shh* expression levels Que et al. (2009) that can take the regulatory system out of Turing parameter space and thereby ensure uniform patterns Kurics et al. (2014). The one-day patterning delay observed in *Fgf10* null mice Sala et al. (2011) may then reflect a delay in the ventral downregulation of *Shh* expression. Apart from chemical signalling, cell-cell interactions can also result in Turing instabilities Watanabe and Kondo (2015). Given the movement and aggregation of *Col2a1*-expressing cells Young et al. (2020), the periodic pattern could, in principle, also arise from chemotaxis Keller and Segel (1971); Hillen and Painter (2009) (Figure 2B), or differential adhesion of cartilage progenitors in the ventral mesenchyme, though additional mechanisms would need to be in place to ensure reproducible stripe formation from noisy initial conditions Armstrong et al. (2006); Canty et al. (2017); Carrillo et al. (2019) (Figure 2C). Finally, differential growth of the ventral epithelium and mesenchyme (Figure 2D) could result in periodic patterning Sultan and Boudaoud (2008); Marin-Riera et al. (2018); Carrillo et al. (2019); Tozluoglu and Mao (2020). Expansion of a thin, incompressible layer with elastic modulus *E*_*Epi*_ and thickness *h* relative to a thick, incompressible substrate with modulus *E*_*Mes*_ results in buckling with wavelength $\lambda =2\pi h{\left(\frac{E_{Epi}}{3E_{Mes}}\right)}^{1/3}$ Sultan and Boudaoud (2008). To obtain the ratio of patterning wavelength, *λ*, and epithelial thickness, *h*, that is observed in the trachea Yoshida et al. (2020), the Young moduli of epithelium, *E*_*Epi*_, and mesenchyme, *E*_*Mes*_, would need to be similar. However, even if the epithelial folds arise from epithelial buckling, they may well be a consequence rather than a driver of mesenchymal condensations. After all, mesenchymal condensations reduce spatial expansion. Given this wide range of possibilities, more quantitative experimental studies and mathematical modelling are required to delineate the mechanism by which the cartilage rings form.

## 7 Conclusion and Outlook

Despite the simplicity of the pattern and the importance of the structure, tracheal cartilage ring formation remains poorly understood. Conditional mutants in combination with explant cultures, organoids, quantitative imaging, and mathematical modelling may help to unravel this patterning mechanism.
